# Targeted genetic and epigenetic profiling of esophageal adenocarcinomas and non-dysplastic Barrett’s esophagus

**DOI:** 10.1186/s13148-022-01287-7

**Published:** 2022-06-14

**Authors:** Rita Pinto, Tobias Hauge, Marine Jeanmougin, Heidi D. Pharo, Stine H. Kresse, Hilde Honne, Sara B. Winge, May-Britt Five, Theresa Kumar, Tom Mala, Truls Hauge, Egil Johnson, Guro E. Lind

**Affiliations:** 1 Department of Molecular Oncology, Institute for Cancer Research, Oslo University Hospital – Norwegian Radium Hospital, Montebello, 0379 Oslo, Norway; 2 K.G. Jebsen Colorectal Cancer Research Centre, Division for Cancer Medicine, Oslo University Hospital, Oslo, Norway; 3 Department of Pediatric and Gastrointestinal Surgery, Oslo University Hospital, Ullevål, Oslo, Norway; 4 Institute of Clinical Medicine, University of Oslo, Oslo, Norway; 5 Department of Pathology, Oslo University Hospital, Ullevål, Oslo, Norway; 6 Department of Gastroenterology, Oslo University Hospital, Ullevål, Oslo, Norway; 7 Department of Biosciences, The Faculty of Mathematics and Natural Sciences, University of Oslo, Oslo, Norway

**Keywords:** Esophageal adenocarcinoma, Barrett’s esophagus, *TP53* mutations, DNA methylation, Microsatellite instability

## Abstract

**Background:**

Despite the efforts to describe the molecular landscape of esophageal adenocarcinoma (EAC) and its precursor lesion Barrett’s esophagus (BE), discrepant findings are reported. Here, we investigated the prevalence of selected genetic (*TP53* mutations and microsatellite instability (MSI) status) and epigenetic (DNA promoter hypermethylation of *APC*, *CDKN2A*, *MGMT*, *TIMP3* and *MLH1*) modifications in a series of 19 non-dysplastic BE and 145 EAC samples. Additional biopsies from adjacent normal tissue were also evaluated. State-of-the-art methodologies and well-defined scoring criteria were applied in all molecular analyses.

**Results:**

Overall, we confirmed frequent *TP53* mutations among EAC (28%) in contrast to BE, which harbored no mutations. We demonstrated that MSI and *MLH1* promoter hypermethylation are rare events, both in EAC and in BE. Our findings further support that *APC*, *CDKN2A*, *MGMT* and *TIMP3* promoter hypermethylation is frequently seen in both lesions (21–89%), as well as in a subset of adjacent normal samples (up to 12%).

**Conclusions:**

Our study further enlightens the molecular background of BE and EAC. To the best of our knowledge, this is one of the largest studies addressing a targeted analysis of genetic and epigenetic modifications simultaneously across a combined series of non-dysplastic BE and EAC samples.

**Supplementary Information:**

The online version contains supplementary material available at 10.1186/s13148-022-01287-7.

## Background

Esophageal cancer is the tenth most commonly diagnosed cancer worldwide causing more than 540,000 deaths annually [1]. Esophagectomy, combined with neoadjuvant radiochemotherapy or chemotherapy, is the mainstay of treatment of resectable tumors. The overall 5-year survival is 20% increasing to nearly 60% in the subgroup of patients undergoing surgery [2]. However, at the time of diagnosis, around 3/4 of the patients are not eligible for surgery due to either too advanced malignant disease or comorbidities.

The two major histological subtypes of esophageal cancer, squamous cell carcinoma and adenocarcinoma (EAC), are characterized by distinct etiologic factors and patterns of incidence and differ not only histologically but also in their underlying molecular characteristics [3]. The incidence of EAC has increased in Western countries, where it currently represents around two-thirds of all esophageal cancers [1]. Most, if not all, EAC arise from a metaplastic lesion termed Barrett’s esophagus (BE), whereby the squamous epithelium of the lower esophagus is replaced by specialized columnar intestinal epithelium, typically as a consequence of chronic gastroesophageal reflux. BE may subsequently progress into EAC through a multistep sequence involving increasing grades of dysplasia [4]. BE is therefore a well-recognized risk factor for the development of EAC, although only a small proportion of patients (< 1%) with non-dysplastic BE develops cancer [5].

Key genetic modifications including chromosomal instability, copy number alterations and mutations have been identified in EAC [6–8]. As for other solid cancer types, the *TP53* tumor suppressor is by far the most recurrently mutated gene in EAC, with reported frequencies from 7 to 83% [6, 9–20]. *TP53* mutations are rarely found in BE with no history of disease progression [7, 21], but they have been reported in dysplastic BE as well as in non-dysplastic BE adjacent to EAC [6, 8].

In addition to genetic aberrations, epigenetic alterations contribute to esophageal malignant transformation and tumor progression. These include histone modifications, aberrant expression of noncoding RNAs and DNA methylation alterations. Hypermethylation of selected gene promoters is observed already during the formation of non-dysplastic BE. Array-based methylation studies support that such DNA methylation changes are early events in EAC development, based on similar aberrations among BE and EAC, which are not found in normal squamous mucosa [22–24]. Among hypermethylated genes in EAC are *APC*, *CDKN2A*, *HPP1*, *RUNX3*, *MGMT* and *TIMP3*, which differ in the reported methylation frequencies [25–33].

In contrast to other gastrointestinal cancers, *MLH1* promoter hypermethylation is infrequent in EAC [26, 34, 35]. Somatic hypermethylation of the *MLH1* promoter with consequent loss of protein expression is the main cause of defective mismatch repair during DNA replication in most sporadic tumors. As mismatch repair defects lead to microsatellite instability (MSI), this condition is, following *MLH1* promoter hypermethylation, expected to be rare in EAC. Only a limited number of studies have addressed MSI status in BE and EAC, reporting inconsistent frequencies [16, 36–41].

Despite the efforts to describe the genetic and epigenetic landscape of EAC, discrepant findings are reported. Many of the studies in the field also rely on the analysis of relatively restricted cohort sizes. In the present study, we have investigated the prevalence of core genetic (*TP53* mutations and MSI status) and epigenetic (DNA promoter hypermethylation of *APC*, *CDKN2A*, *MGMT*, *TIMP3* and *MLH1*) modifications in a cohort of non-dysplastic BE and a large series of EAC samples.

## Results

**Fig. 1:**
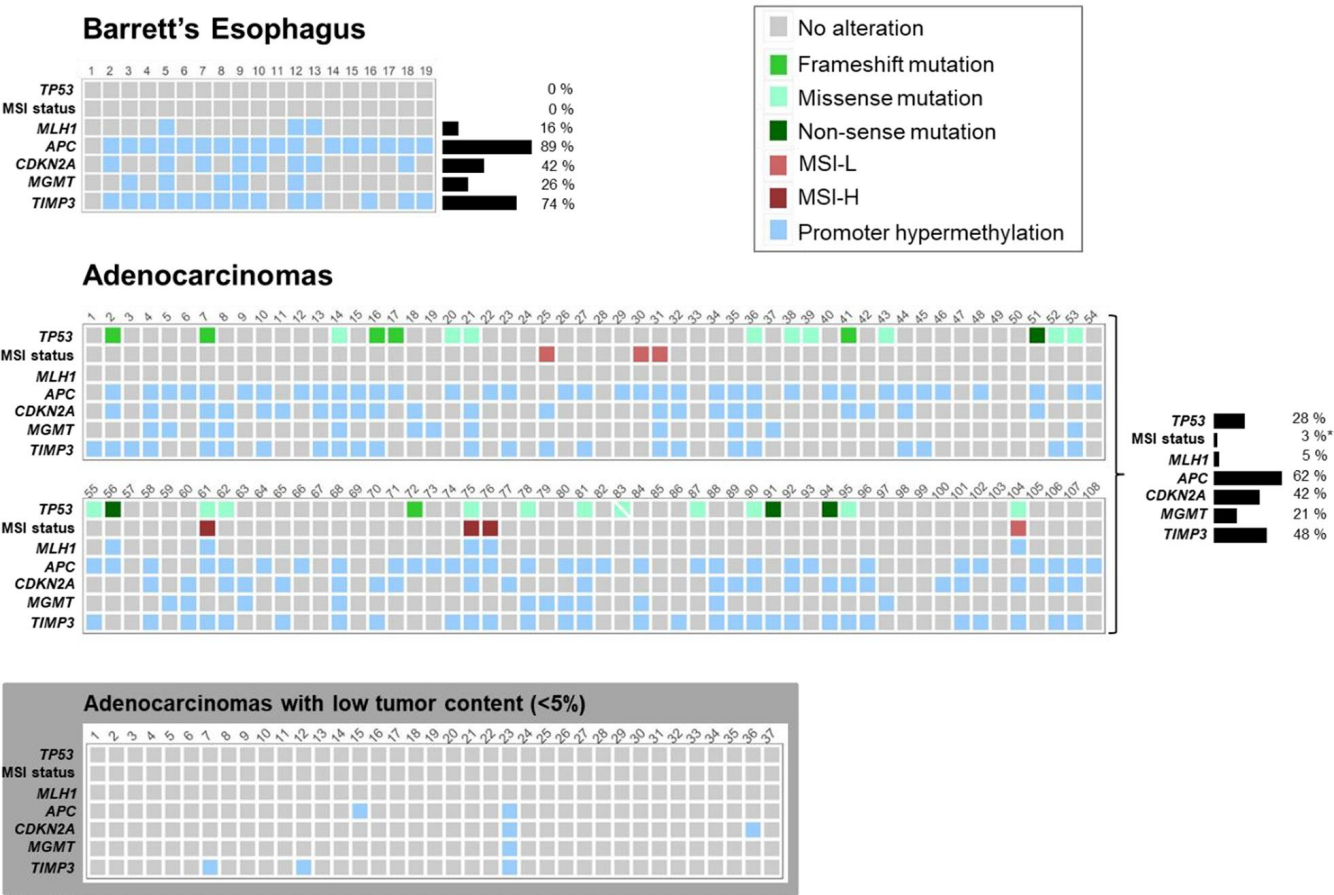
Summary of genetic and epigenetic alterations in BE (*n* = 19) and EAC (*n* = 145) samples. *TP53* silent mutations with no amino acid change are not presented as alterations. In one sample (patient 83), two *TP53* missense mutations were found. All samples with no or a low percentage of tumor cells (< 5%; *n* = 37) have been removed from the main data set (see Fig. 2), and the molecular alterations found in these samples are shown separately in the gray box. These samples were not used for determination of alterations frequencies. (*For MSI, the percentage refers to MSI-H tumors only.)

**Fig. 2:**
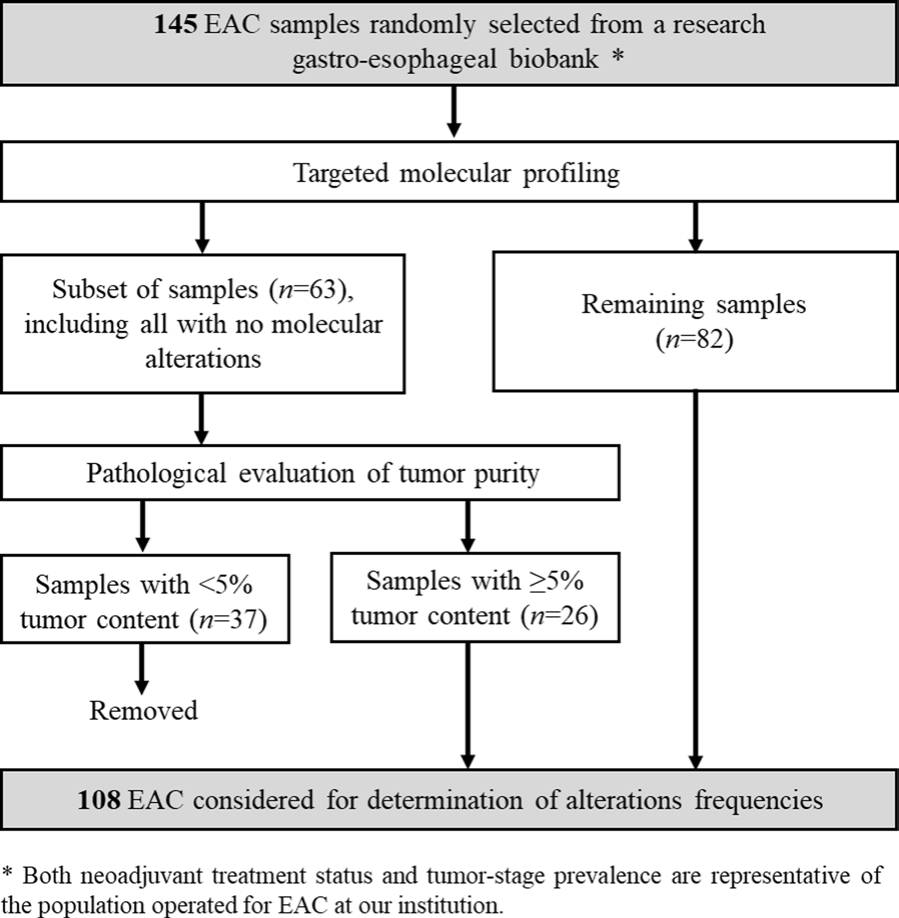
Flow diagram illustrating EAC samples selection process. One hundred and forty-five EAC patients were subjected to targeted molecular profiling, among which 37 were removed from the main data set due to the absence of tumor or low tumor cell content (< 5%). Only samples from 108 patients were used for determination of alterations frequencies

An overview of the results is shown in Fig. 1, and detailed information about the sample selection process is illustrated in Fig. 2.

### Frequency, location and type of *TP53* mutations

**Fig. 3:**
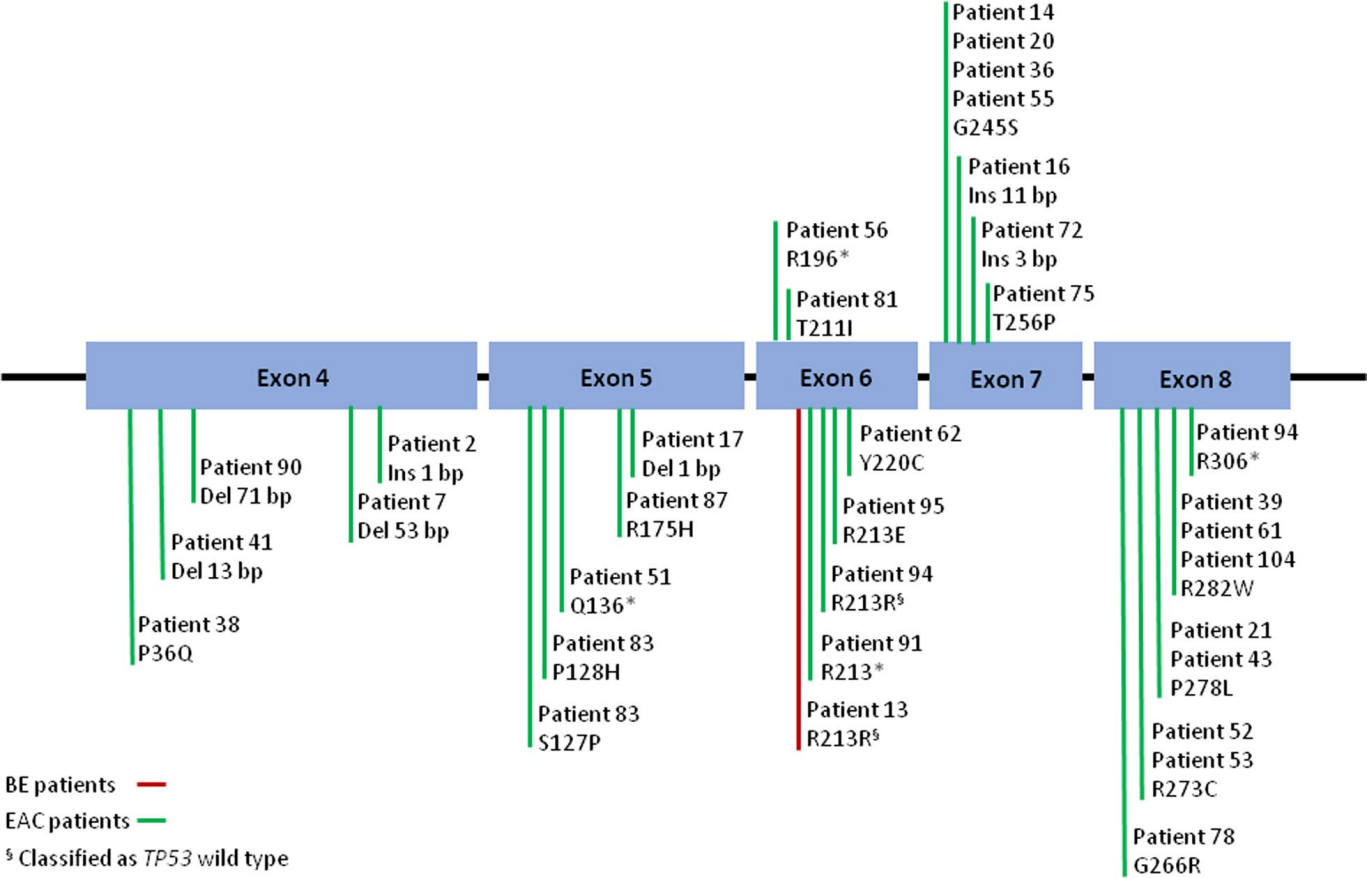
Graphical representation of *TP53* mutations identified in BE and EAC samples. The entire *TP53* coding region (exons 2–11) was analyzed by Sanger sequencing, and mutations were found across exons 4–8. The silent mutation R213R found in one BE (patient 13) and one EAC (patient 94) sample was classified as *TP53* wild type

*TP53* mutations detected in BE and EAC are shown in Fig. 3 and listed in Additional file 1: Table S1. A silent mutation with no amino acid change was detected in one BE (patient 13), which was classified as *TP53* wild type and therefore not considered as a mutation hereafter. The same silent mutation was detected in one EAC (patient 94), but this sample was still considered altered due to the presence of another *TP53* mutation.

Overall, none of the BE harbored *TP53* mutations, whereas 30 out of 108 (28%) EAC samples carried mutations. One of the EAC samples (patient 83) harbored two different mutations. Seven of a total of 31 mutations (23%) were indels, while the rest were point mutations leading to amino acid substitution (missense mutation), four of them involving a stop codon (nonsense mutations). The 31 *TP53* gene mutations were distributed as follows: five in exon 4, five in exon 5, five in exon 6, seven in exon 7 and nine in exon 8. No mutation was found in exons 2–3 or 9–11. G:C to A:T single-base transitions were predominant among point mutations (21 out of 24 mutations, 88%), eleven of which occurred at CpG dinucleotides.

A significant association was observed between *TP53* mutations and increased age (*p* = 0.021, Wilcoxon’s test) and between *TP53* mutations and gender (*p* = 0.0027, Fisher’s exact test). In addition, neoadjuvant treatment of EAC patients was found to be significantly associated with the absence of *TP53* mutations (*p* = 0.045, Fisher’s exact test; Additional file 1: Table S2). Age is associated with the decision of treating patients with neoadjuvant therapy (*p* = 3.4 × 10^–10^). As age is a confounding factor when testing for potential association between *TP53* mutation and neoadjuvant treatment, the patients were stratified into two subgroups, > 75 (*n* = 20) and ≤ 75 years old (*n* = 88). No significant association was found between *TP53* mutations and neoadjuvant treatment in these subgroups.

### MSI status and *MLH1* promoter hypermethylation

**Fig. 4:**
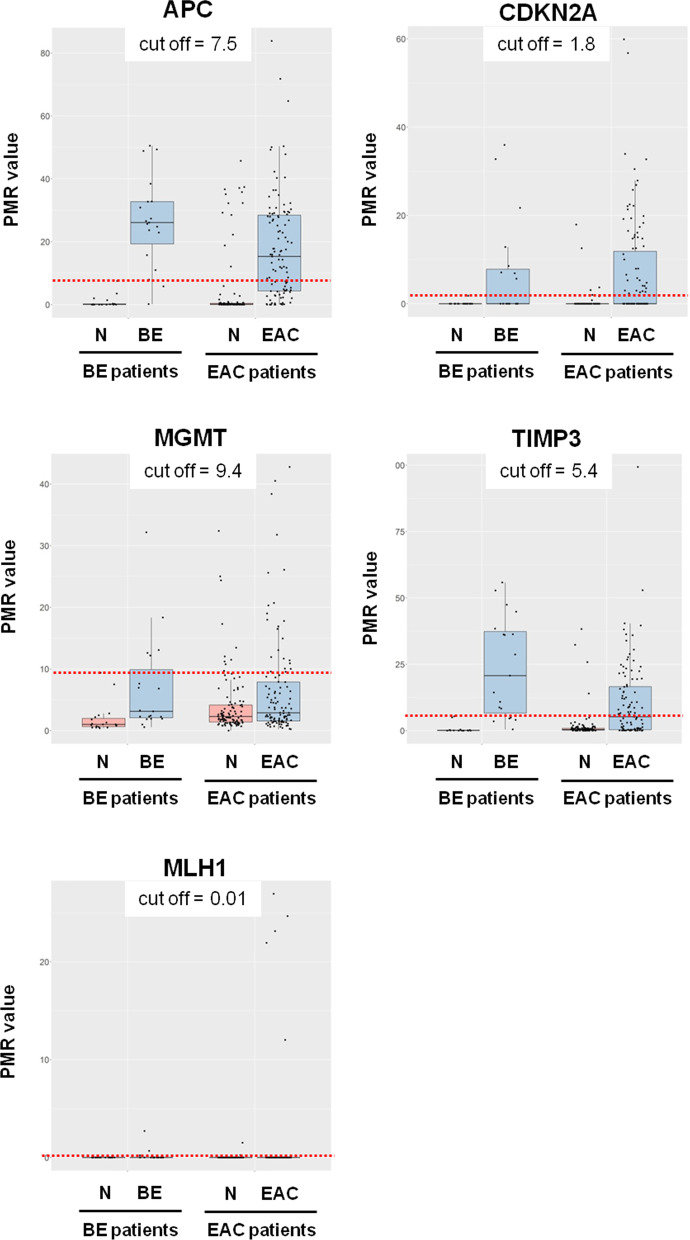
PMR values distribution in BE (*n* = 19), EAC (*n* = 108) and respective normal adjacent mucosa (N). The thresholds for scoring the samples as methylated were set according to the highest PMR value across the normal mucosa matching BE samples. These thresholds were determined for each gene independently and are marked by dotted red lines

None of the BE lesions and seven out of 108 tumors (6%) showed MSI. Of the MSI tumors, three were scored as having high degree of MSI (MSI-H) and four as having low degree of MSI (MSI-L). All three MSI-H tumors had hypermethylated *MLH1* promoters (*p* = 4.9 × 10^–5^, Fisher’s exact test). Among the microsatellite stable (MSS) samples, three BE (16%) and one EAC (1%) showed *MLH1* promoter hypermethylation. Methylation frequencies are shown in Fig. 1 for BE and EAC samples, and in Additional file 1: Table S3 for normal samples matching EAC. The distribution of *MLH1* PMR values is illustrated in Fig. 4. No significant associations were found between MSI-H status or *MLH1* promoter hypermethylation and clinicopathological data.

### Promoter methylation frequencies of *APC*, *CDKN2A*, *MGMT* and *TIMP3*

We examined the promoter DNA methylation status of four genes (*APC*, *CDKN2A*, *MGMT* and *TIMP3*) in addition to *MLH1*. The distribution of PMR values is illustrated in Fig. 4. A subset of normal samples adjacent to EAC (up to 12%) harbored promoter hypermethylation. For each gene, the promoter methylation frequency was significantly higher in BE or tumor samples (Fig. 1) compared to the tumor adjacent normal counterpart (Additional file 1: Table S3) (*p* < 0.05, Fisher’s exact test if BE *vs* normal and McNemar’s test if EAC *vs* normal). Three BE (16%) and 8 EAC (7%) samples showed hypermethylation of all four genes simultaneously (Fig. 1).

**Fig. 5:**
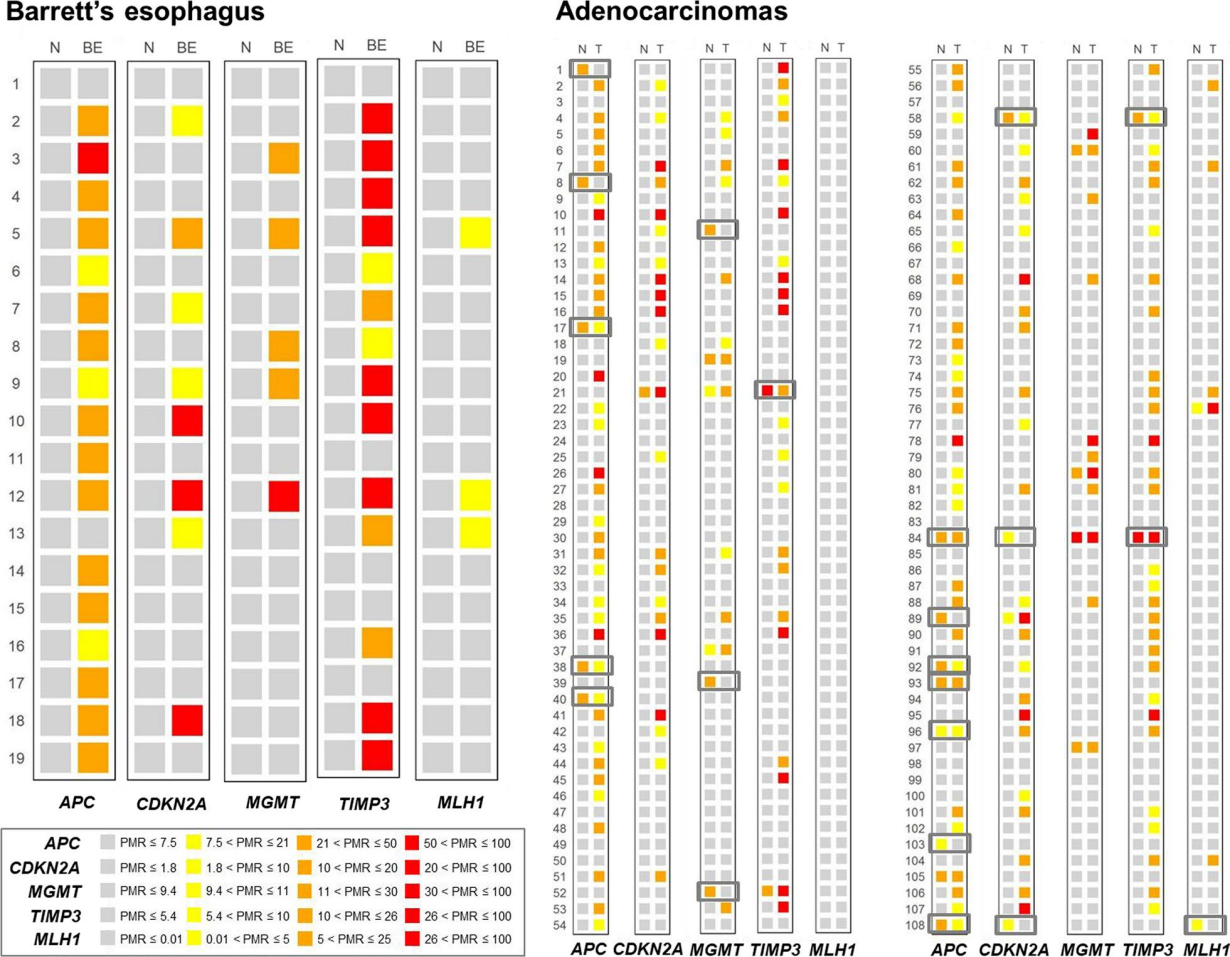
Methylation levels of the evaluated genes in BE, EAC (T) and normal adjacent mucosa (N). EAC patients where normal mucosa presents PMR values higher than in the tumor are highlighted by gray bordered boxes. PMR values are shown in different color scales for each gene in order to facilitate visualization

The PMR values for individual genes in BE and EAC patients are shown in Fig. 5. Seventeen EAC patients (16%) had lower PMR values in the tumor compared with the matching adjacent mucosa for at least one gene. Nine of them (8% of all EAC patients) presented promoter hypermethylation in adjacent mucosa but not in the tumor for one or two of the genes. All but one of these pairs had other aberrations (mutations or hypermethylation) in the tumor sample, and pentanucleotide marker controls included in MSI analysis confirmed that EAC samples and normal counterparts belonged to the same patient. A single tumor presented no alterations (patient 103) despite a 20–30% tumor cell content.

A significant association was observed between *APC* promoter hypermethylation and male gender (*p* = 0.035, Fisher’s exact test) in BE patients. In EAC patients, a significant association was found between *CDKN2A*, *MGMT* or *TIMP3* promoter hypermethylation and tumor location (*p* = 0.034, *p* = 0.0070 and *p* = 0.013, respectively, Fisher’s exact test). In addition, a significant association was observed between *TIMP3* and age (*p* = 0.036, Wilcoxon’s test) or tumor stage (*p* = 0.011, Fisher’s exact test). The use of neoadjuvant treatment and the absence of *CDKN2A* or *TIMP3* promoter methylation were also found to be statistically associated (*p* = 0.043 and *p* = 0.0034, respectively, Fisher’s exact test; Additional file 1: Table S2) when including all patients. However, these associations did not remain significant when patients were stratified by age.

## Discussion

Description of molecular alterations in EAC is abundant in the literature, but discrepancies regarding frequency of these alterations have been observed across studies. In the present work, we analyzed key molecular features in a cohort of non-dysplastic BE and a large series of EAC tissue samples using robust methodologies and well-defined scoring criteria. Overall, our results confirmed frequent *TP53* mutations among EAC in contrast to non-dysplastic BE lesions, which harbored no mutations. Our findings also support that promoter hypermethylation is an early event in the multistep progression of EAC and frequently seen in BE. Finally, we demonstrated that MSI and *MLH1* promoter hypermethylation are rare events in both lesions.

The series of EAC samples analyzed here was selected to be representative of the population operated for EAC at our institution, in terms of both neoadjuvant treatment status and of tumor-stage prevalence. As expected, many of the EAC samples with no detected molecular alterations had no or low tumor cell content (< 5%; *n* = 32), demonstrating the value of histopathological evaluation. These samples were left out when mutation and methylation frequencies were calculated, but otherwise kept in order to report the unbiased results of a representative series. Among the cases not evaluated by histopathology, but with one or more molecular alterations (Fig. 2), the percentage of EAC samples with no or low tumor cell content would be expected to be lower than the evaluated cases. However, we cannot rule out that some of these EACs might have a lower tumor percentage than the limit of detection of the various molecular analyses, potentially lowering the frequencies reported.

Among molecular abnormalities in EAC, mutation of *TP53* tumor suppressor is one of the most common. We detected *TP53* mutations in 28% of the tumors, while most of the previous studies reported mutation frequencies above 40% [6, 9–11, 13, 16, 17, 20]. Some of this mutation frequency discrepancy may be explained by treatment status. In the present study, we found that tumors from treatment-naïve patients had 44% *TP53* mutations, which is closer to the frequencies reported in other studies including treatment-naïve patients only [11, 16]. In contrast, neoadjuvant treated tumors harbored only half as many mutations. The lower *TP53* mutation frequency reported here may therefore be an effect of the sample series composition. In addition, we cannot exclude that some *TP53* mutations may have been missed due to the limit of detection in Sanger sequencing analyses. Although the number of studies on non-dysplastic BE is more limited, *TP53* mutations have been detected in this lesion when resected from tissue adjacent to the tumor [6, 8], while they are rarely found in non-dysplastic BE of patients who have never developed cancer [7, 21]. In line with these observations, *TP53* mutations were not found in our series of non-dysplastic BE samples.

Here, all exons constituting the coding region of the canonical p53 protein (exons 2–11) were covered. Most of the previous studies span only exons 5–8, the region coding for p53 DNA-binding domain. However, although rare, mutations outside this region and in particular in exon 4 occur in EAC [11, 13, 20], as well as in other cancer types [42]. In the present study, 16% of the detected mutations were found in exon 4. These findings demonstrate the importance of analyzing regions outside exons 5–8 and suggest that mutations of *TP53* in exon 4 may also play a role in EAC development. To the best of our knowledge, all of the point mutations identified in our study were previously described in EAC [10–13, 15, 17, 19, 20, 43], with the exception of S127P, P128H (both in patient 83), Q136* (patient 51), T211I (patient 81) and Y220C (patient 62). Codon 220 has been reported as a “hotspot” for *TP53* mutations in other types of cancers [42].

MSI has also been investigated in EAC by others. Differences in the number and nature of the evaluated markers, as well as in scoring criteria, may contribute to discrepancies in MSI prevalence observed across studies [16, 36–41]. Based on the markers recommended by the National Cancer Institute [36, 37, 39, 41], we found no BE MSI cases and low MSI-H frequency in EAC (3%). In sporadic colorectal cancer, the MSI phenotype is associated with *MLH1* promoter hypermethylation, which is the most common mechanism of *MLH1* silencing in this cancer type [44]. These events have also been related in EAC [34, 37]. Here, we observed a low frequency of *MLH1* hypermethylation (5%), in agreement with the low prevalence of MSI. We further showed that *MLH1* promoter is hypermethylated in all MSI-H cases. On the other hand, only one of the *MLH1* hypermethylated tumors was MSS, in line with the small fraction (< 10%) observed in sporadic colorectal tumors [44, 45]. Interestingly, among the samples with *MLH1* promoter hypermethylation, *MLH1* PMR values were considerably lower in BE samples than in EAC. This may reflect the pre-neoplastic nature of BE lesions. Since all of the BE samples were scored as MSS, they clearly have a functioning mismatch repair system, indicating that the reported promoter methylation level for *MLH1* was not high enough to inactivate it.

We showed frequent promoter hypermethylation for most genes both in non-dysplastic BE and in EAC. In BE, these observations may be a consequence of the prolonged exposure to gastroesophageal reflux, causing an inflammatory environment and tissue damage, often related to epigenetic alterations. Similarities in the methylation profiles of BE and EAC have been documented in several array-based methylation studies, including both non-dysplastic and dysplastic BE [22–24]. Interestingly, promoter methylation frequencies were higher in BE than in EAC for all genes except *CDKN2A*. Such high methylation frequencies in non-dysplastic BE samples have also been previously reported by others [35].

We also detected promoter hypermethylation in a subset of normal samples adjacent to EAC, as previously reported in histologically normal tissues adjacent to EAC [9, 25, 28, 30]. Notably, the highest methylation frequencies in normal mucosa were observed for *APC* and *MGMT*, two markers of field defect in prostate [46] and sporadic colorectal cancers, [47], respectively. For some of the EAC patients, lower PMR values were detected in the tumor sample than in the normal counterpart. In the case of *APC* and *CDKN2A*, these findings may in part reflect the deletion of the methylated alleles attributable to loss of heterozygosity, which has been reported in EAC [6, 17, 18].

We have found a statistically significant association between the use of neoadjuvant treatment in EAC patients and the absence of mutations in *TP53* or methylation of specific genes (*CDKN2A* or *TIMP)*. Moreover, 92% of the EAC patients showing no alterations across the set of markers had received neoadjuvant treatment. When stratifying patients by age, these associations lost their significance as age is a confounder of treatment. These observations are in line with a previous analysis of the DNA methylation patterns in EAC patients, which revealed no differences between patients receiving neoadjuvant chemotherapy or not [48], regardless of age. Additional studies analyzing potential associations between neoadjuvant treatment and genomic or molecular aberrations in EACs are warranted.

In total, 13 tumor samples (12%) showed no alterations—including mutations or hypermethylation. It could be speculated that these samples correspond to lower stages of the disease. We have selected our set of samples based on the representativity of tumor-stage prevalence in EAC patients eligible for surgery, and therefore it inevitably comprises a low percentage of stage IV tumors. Nevertheless, we found no association between the absence of alterations and tumor stage (*p* = 0.56, Fisher’s exact test).

Overall, the prevalence of *TP53* mutations, as well as promoter methylation frequency of *APC*, *CDKN2A*, *MGMT* and *TIMP3*, observed in EAC shows some discrepancies when compared to findings reported in other studies. Our results are based on a sample size larger than most of these, adding another layer of robustness to our analyses. Factors that may explain the inconsistencies in the methylation results may include differences in the prevalence of tumor location (distal esophagus *vs* gastroesophageal junction) and the thresholds used for distinction between methylated and unmethylated DNA. We have here considered normal mucosa adjacent to BE samples as “methylation background” and defined the threshold for each gene individually.

## Conclusions

The present study contributes to an improved characterization of the molecular background of EAC progression by analyzing a series of non-dysplastic BE, EAC and matched normal samples. We reported a spectrum of genetic and epigenetic alterations occurring in these tissues and clarified discrepancies found in the literature regarding frequency of these alterations. Our study derived its strength from a careful design, use of consensus markers, state-of-the-art methodologies and well-defined scoring criteria. To the best of our knowledge, this is one of the largest studies addressing a targeted characterization of genetic and epigenetic modifications simultaneously across a combined series of non-dysplastic BE and EAC samples.

## Methods

### Patients and tumor samples

This study included tissue samples from 19 BE patients without a current dysplasia or a history of dysplasia and from 145 EAC patients. BE biopsies were collected between November 2017 and February 2020 during routine gastroscopy at the Department of Gastroenterology, Oslo University Hospital, Ullevål. BE was defined as the presence of columnar epithelium in the distal esophagus containing specialized intestinal metaplasia with a minimum length of 1 cm [49]. Four-quadrant biopsies were taken every 2 cm within BE segment, in accordance with the current guidelines. Among these, multiple (2–4) samples were randomly chosen to be used in this study and pooled for DNA extraction. EAC samples were obtained from patients operated between September 2013 and May 2020 at the Department of Pediatric and Gastrointestinal Surgery, Oslo University Hospital, Ullevål. One hundred and seventeen (81%) EAC patients had received neoadjuvant radio(chemo)therapy. Only patients with macroscopic residual tumor left in the surgical specimen (stages T0–T4) were included in this study. Both neoadjuvant treatment status and tumor-stage prevalence are representative of the population operated for EAC at our institution [50]. For all patients (*n* = 164), matched biopsies from adjacent macroscopically normal-appearing mucosa (5–10 cm from the tumor), hereafter referred to as normal samples, were included. Samples were taken immediately following specimen resection according to a predefined protocol. For all the paired BE and normal samples, as well as for 103 (71%) of the paired EAC and normal counterparts, patient identity was verified by short tandem repeat (STR) profiling according to the AmpFLSTR Identifiler PCR Amplification Kit (Thermo Fisher Scientific). Clinicopathological characteristics of the EAC patients are summarized in Additional file 1: Table S4.

Sixty-three EAC samples were subjected to histopathological evaluation as described in Fig. 2. Of these, 37 were removed from frequency calculations due to the absence of tumor or low tumor cell content (< 5%). The main series of this study therefore comprised samples from 108 patients (Fig. 1). Clinicopathological characteristics of these patients are summarized in Table 1.

**Table 1:** Summary of clinicopathological characteristics of patient samples considered in this study after removal of the samples with no or a low percentage of tumor cells (< 5%)

	BE (*n* = 19)	EAC (*n* = 108)
Age (years)		
Median (mean)	66 (62)	66 (66)
Range	35–84	34–82
Gender		
Male	15 (79%)	90 (83%)
Female	4 (21%)	18 (17%)
Barrett’s segment length (cm)		
Median (mean)	4 (4.4)	–
Range	1–10	–
Location		
At or above carina	–	1 (1%)
Distal esophagus	–	37 (34%)
Gastroesophageal junction	–	70 (65%)
Tumor (T) stage		
T0	–	1 (1%)
T1	–	16 (15%)
T2	–	20 (18%)
T3	–	69 (64%)
T4	–	2 (2%)
Tumor length (cm)		
Median (mean)	–	3.0 (3.6)
Range	–	0.4–11
Lymph node metastases		
Yes	–	65 (60%)
No	–	43 (40%)
Neoadjuvant radio(chemo)therapy		
Yes	–	81 (75%)
No	–	27 (25%)

### DNA extraction and bisulfite treatment

DNA from fresh frozen tissue samples corresponding to tumors and matched normal mucosa was extracted using the DNeasy Blood and Tissue Kit (Qiagen). For samples from BE biopsies (< 30 mg) and adjacent normal mucosa, the AllPrep DNA/RNA Mini Kit (Qiagen) was used. DNA quantity and quality were measured using ND-1000 Nanodrop (NanoDrop Technologies). For the methylation analyses, 800 ng DNA of each sample was bisulfite-treated using the EpiTect Bisulfite Kit (Qiagen) according to the manufacturer’s protocol. Bisulfite-converted DNA was purified using the QIAcube automated pipetting system (Qiagen) and eluted in 40 μl elution buffer.

### Selection of candidate genes for analysis

A literature search was performed in order to identify candidate genes in EAC (Additional file 1: Figure S1). Genes consistently reported as frequently altered (> 50%) in at least three original papers were considered for inclusion. Based on this search, *TP53* was selected for mutation analysis, whereas *APC*, *CDKN2A*, *MGMT* and *TIMP3* were selected for DNA methylation analysis. In addition, *MLH1* promoter methylation, reported to be infrequent in EAC, was analyzed in order to relate it to MSI status.

### *TP53* mutation analysis

*TP53* mutation status was assessed in all BE and EAC samples by Sanger sequencing. The entire coding region (exons 2–11) was analyzed using previously described primer sequences and reactions [51]. Mutation calling was performed independently by two of the authors, using the SeqScape V.2.5 and Sequencing Analysis V.5.3.1 software (both Applied Biosystems). All detected mutations were confirmed by sequencing of a new independent PCR product.

### Microsatellite instability analysis

MSI status was assessed in all BE and EAC and compared with corresponding normal tissue by PCR-based analyses of the BAT-25, BAT-26, NR-21, NR-24 and MONO-27 mononucleotide markers using the MSI Analysis System, Version 1.2 (Promega) according to the manufacturer’s instructions. Data were analyzed with GeneMapper software (Applied Biosystems). Nuclease-free water replacing DNA as template was included in each run as control. All the paired samples (BE or EAC and normal counterparts) were confirmed to belong to the same patient by analyzing pentanucleotide marker controls available in the MSI Analysis System.

The results were scored independently by two of the authors following Bethesda guidelines for colorectal cancer [52]. MSI-H in BE or tumor DNA was defined if two or more markers showed aberrant peak profile, whereas one single unstable marker defined MSI-L. Samples with all loci exhibiting normal allelic ranges were regarded MSS. MSI status for each locus was confirmed by an independent run.

### Quantitative methylation-specific PCR

*APC*, *CDKN2A*, *MGMT*, *TIMP3* and *MLH1* were analyzed for DNA promoter hypermethylation in all BE, EAC and adjacent normal samples using quantitative methylation-specific PCR (qMSP) and *ALU-C4* as a normalization control for DNA input. Primer and probe sequences have been reported previously [35, 53]. Primers were purchased from BioNordika (Oslo, Norway), and probes were obtained from Life Technologies (now Thermo Fisher Scientific).

The qMSP reactions were performed in triplicate and carried out as previously described [54] using ~ 30 ng bisulfite-treated DNA per well. Methylation-positive (in vitro methylated DNA; IVD Chemicon, Millipore), methylation-negative (WGA non-methylated DNA; Zymo Research) and non-template (H_2_O) controls were included, in addition to a standard curve consisting of a fivefold serial dilution of IVD (32.5–0.052 ng).

Samples amplified after cycle 35 were censored in accordance with the recommendations from Life Technologies, and the median quantity value of the triplicates was used for data analysis. The qMSP results were calculated as percent of methylated reference (PMR) by dividing the *ALU-C4*-normalized quantity of the samples by the *ALU-C4*-normalized quantity of the positive control (IVD) and multiply by 100. To ensure high specificity for each qMSP assay, the thresholds for scoring samples as methylated were set according to the highest PMR value across the normal mucosa matching BE samples as shown in Fig. 4. Samples with PMR values above the scoring threshold for each individual gene were considered to be methylated.

#### Statistics

Statistical analyses were conducted with R software version 3.6.2. Associations between gene alterations and the clinicopathological parameters listed in Table 1 were analyzed by Fisher’s exact tests for categorical variables and by two-sided Wilcoxon’s tests for continuous variables. Associations between genetic and epigenetic alterations were investigated using Fisher’s exact tests or McNemar’s tests. A *p* value < 0.05 was considered significant. When relevant, *p* values were adjusted for multiple testing using the FDR criterion and Benjamini–Hochberg procedure. An adjusted *p* value < 0.05 was considered significant.

## Supplementary Information

**Additional file 1: Tables S1, S2, S3, and S4. **Detailed list of TP53 mutations identified in BE and EAC samples; Summary of the genetic and epigenetic alterations found in neoadjuvant treatment-naïve and treated EAC patients; Promoter methylation frequencies in normal mucosa adjacent to EAC samples; and summary of clinicopathological characteristics of all included EAC patient samples.

**Additional file 2: Figure S1** Literature search flow diagram for selection of candidate genes for analysis.

## Data Availability

The data sets used and/or analyzed during the current study are available from the corresponding author on reasonable request.

## Abbreviations

BE: Barrett’s esophagus
EAC: Esophageal adenocarcinoma
IVD: In vitro methylated DNA
MSI: Microsatellite instability
MSI-H: High degree of microsatellite instability
MSI-L: Low degree of microsatellite instability
MSS: Microsatellite stable
PMR: Percent of methylated reference
qMSP: Quantitative methylation-specific PCR
STR: Short tandem repeat

## References

[1] Sung H, Ferlay J, Siegel RL, Laversanne M, Soerjomataram I, Jemal A (2021). Global cancer statistics 2020: GLOBOCAN estimates of incidence and mortality worldwide for 36 cancers in 185 countries. CA Cancer J Clin.

[2] Hauge T, Forland DT, Johannessen HO, Johnson E (2021). Short- and long-term outcomes in patients operated with total minimally invasive esophagectomy for esophageal cancer. Dis Esophagus.

[3] Smyth EC, Lagergren J, Fitzgerald RC, Lordick F, Shah MA, Lagergren P (2017). Oesophageal cancer. Nat Rev Dis Primers.

[4] Flejou JF (2005). Barrett's oesophagus: from metaplasia to dysplasia and cancer. Gut.

[5] Desai TK, Krishnan K, Samala N, Singh J, Cluley J, Perla S (2012). The incidence of oesophageal adenocarcinoma in non-dysplastic Barrett's oesophagus: a meta-analysis. Gut.

[6] Ross-Innes CS, Becq J, Warren A, Cheetham RK, Northen H, O'Donovan M (2015). Whole-genome sequencing provides new insights into the clonal architecture of Barrett's esophagus and esophageal adenocarcinoma. Nat Genet.

[7] Stachler MD, Camarda ND, Deitrick C, Kim A, Agoston AT, Odze RD (2018). Detection of mutations in Barrett's esophagus before progression to high-grade dysplasia or adenocarcinoma. Gastroenterology.

[8] Stachler MD, Taylor-Weiner A, Peng S, McKenna A, Agoston AT, Odze RD (2015). Paired exome analysis of Barrett's esophagus and adenocarcinoma. Nat Genet.

[9] Baumann S, Keller G, Puhringer F, Napieralski R, Feith M, Langer R (2006). The prognostic impact of O6-Methylguanine-DNA Methyltransferase (MGMT) promotor hypermethylation in esophageal adenocarcinoma. Int J Cancer.

[10] Bian YS, Osterheld MC, Bosman FT, Benhattar J, Fontolliet C (2001). p53 gene mutation and protein accumulation during neoplastic progression in Barrett's esophagus. Mod Pathol.

[11] Casson AG, Evans SC, Gillis A, Porter GA, Veugelers P, Darnton SJ (2003). Clinical implications of p53 tumor suppressor gene mutation and protein expression in esophageal adenocarcinomas: results of a ten-year prospective study. J Thorac Cardiovasc Surg.

[12] Casson AG, Mukhopadhyay T, Cleary KR, Ro JY, Levin B, Roth JA (1991). p53 gene mutations in Barrett's epithelium and esophageal cancer. Cancer Res.

[13] Chung SM, Kao J, Hyjek E, Chen YT (2007). p53 in esophageal adenocarcinoma: a critical reassessment of mutation frequency and identification of 72Arg as the dominant allele. Int J Oncol.

[14] Djalilvand A, Pal R, Goldman H, Antonioli D, Kocher O (2004). Evaluation of p53 mutations in premalignant esophageal lesions and esophageal adenocarcinoma using laser capture microdissection. Mod Pathol.

[15] Dolan K, Walker SJ, Gosney J, Field JK, Sutton R (2003). TP53 mutations in malignant and premalignant Barrett's esophagus. Dis Esophagus.

[16] Evans SC, Gillis A, Geldenhuys L, Vaninetti NM, Malatjalian DA, Porter GA (2004). Microsatellite instability in esophageal adenocarcinoma. Cancer Lett.

[17] Gleeson CM, Sloan JM, McGuigan JA, Ritchie AJ, Russell SE (1995). Base transitions at CpG dinucleotides in the p53 gene are common in esophageal adenocarcinoma. Cancer Res.

[18] Gonzalez MV, Artimez ML, Rodrigo L, Lopez-Larrea C, Menendez MJ, Alvarez V (1997). Mutation analysis of the p53, APC, and p16 genes in the Barrett's oesophagus, dysplasia, and adenocarcinoma. J Clin Pathol.

[19] Novotna K, Trkova M, Pazdro A, Smejkal M, Soukupova A, Kodetova D (2006). TP53 gene mutations are rare in nondysplastic Barrett's esophagus. Dig Dis Sci.

[20] Schneider PM, Stoeltzing O, Roth JA, Hoelscher AH, Wegerer S, Mizumoto S (2000). P53 mutational status improves estimation of prognosis in patients with curatively resected adenocarcinoma in Barrett's esophagus. Clin Cancer Res.

[21] Weaver JMJ, Ross-Innes CS, Shannon N, Lynch AG, Forshew T, Barbera M (2014). Ordering of mutations in preinvasive disease stages of esophageal carcinogenesis. Nat Genet.

[22] Xu E, Gu J, Hawk ET, Wang KK, Lai M, Huang M (2013). Genome-wide methylation analysis shows similar patterns in Barrett's esophagus and esophageal adenocarcinoma. Carcinogenesis.

[23] Krause L, Nones K, Loffler KA, Nancarrow D, Oey H, Tang YH (2016). Identification of the CIMP-like subtype and aberrant methylation of members of the chromosomal segregation and spindle assembly pathways in esophageal adenocarcinoma. Carcinogenesis.

[24] Jammula S, Katz-Summercorn AC, Li X, Linossi C, Smyth E, Killcoyne S (2020). Identification of subtypes of Barrett's esophagus and esophageal adenocarcinoma based on DNA methylation profiles and integration of transcriptome and genome data. Gastroenterology.

[25] Brock MV, Gou M, Akiyama Y, Muller A, Wu TT, Montgomery E (2003). Prognostic importance of promoter hypermethylation of multiple genes in esophageal adenocarcinoma. Clin Cancer Res.

[26] Clement G, Braunschweig R, Pasquier N, Bosman FT, Benhattar J (2006). Methylation of APC, TIMP3, and TERT: a new predictive marker to distinguish Barrett's oesophagus patients at risk for malignant transformation. J Pathol.

[27] Eads CA, Lord RV, Kurumboor SK, Wickramasinghe K, Skinner ML, Long TI (2000). Fields of aberrant CpG island hypermethylation in Barrett's esophagus and associated adenocarcinoma. Cancer Res.

[28] Hardie LJ, Darnton SJ, Wallis YL, Chauhan A, Hainaut P, Wild CP (2005). p16 expression in Barrett's esophagus and esophageal adenocarcinoma: association with genetic and epigenetic alterations. Cancer Lett.

[29] Sarbia M, Geddert H, Klump B, Kiel S, Iskender E, Gabbert HE (2004). Hypermethylation of tumor suppressor genes (p16INK4A, p14ARF and APC) in adenocarcinomas of the upper gastrointestinal tract. Int J Cancer.

[30] Schulmann K, Sterian A, Berki A, Yin J, Sato F, Xu Y (2005). Inactivation of p16, RUNX3, and HPP1 occurs early in Barrett's-associated neoplastic progression and predicts progression risk. Oncogene.

[31] Smith E, De Young NJ, Pavey SJ, Hayward NK, Nancarrow DJ, Whiteman DC (2008). Similarity of aberrant DNA methylation in Barrett's esophagus and esophageal adenocarcinoma. Mol Cancer.

[32] Vieth M, Schneider-Stock R, Rohrich K, May A, Ell C, Markwarth A (2004). INK4a-ARF alterations in Barrett's epithelium, intraepithelial neoplasia and Barrett's adenocarcinoma. Virchows Arch.

[33] Wong DJ, Barrett MT, Stoger R, Emond MJ, Reid BJ (1997). p16INK4a promoter is hypermethylated at a high frequency in esophageal adenocarcinomas. Cancer Res.

[34] Geddert H, Kiel S, Iskender E, Florl AR, Krieg T, Vossen S (2004). Correlation of hMLH1 and HPP1 hypermethylation in gastric, but not in esophageal and cardiac adenocarcinoma. Int J Cancer.

[35] Eads CA, Lord RV, Wickramasinghe K, Long TI, Kurumboor SK, Bernstein L (2001). Epigenetic patterns in the progression of esophageal adenocarcinoma. Cancer Res.

[36] Cancer Genome Atlas Research N, Analysis Working Group: Asan U, Agency BCC, Brigham, Women's H, Broad I, et al. Integrated genomic characterization of oesophageal carcinoma. Nature. 2017;541(7636):169–75. 10.1038/nature20805 PMC5651175 28052061

[37] Farris AB, Demicco EG, Le LP, Finberg KE, Miller J, Mandal R (2011). Clinicopathologic and molecular profiles of microsatellite unstable Barrett esophagus-associated adenocarcinoma. Am J Surg Pathol.

[38] Gleeson CM, Sloan JM, McGuigan JA, Ritchie AJ, Weber JL, Russell SE (1996). Ubiquitous somatic alterations at microsatellite alleles occur infrequently in Barrett's-associated esophageal adenocarcinoma. Cancer Res.

[39] Kulke MH, Thakore KS, Thomas G, Wang H, Loda M, Eng C (2001). Microsatellite instability and hMLH1/hMSH2 expression in Barrett esophagus-associated adenocarcinoma. Cancer.

[40] Muzeau F, Flejou JF, Belghiti J, Thomas G, Hamelin R (1997). Infrequent microsatellite instability in oesophageal cancers. Br J Cancer.

[41] Shiraishi H, Mikami T, Yoshida T, Tanabe S, Kobayashi N, Watanabe M (2006). Early genetic instability of both epithelial and stromal cells in esophageal squamous cell carcinomas, contrasted with Barrett's adenocarcinomas. J Gastroenterol.

[42] Hainaut P, Pfeifer GP (2016). Somatic TP53 mutations in the era of genome sequencing. Cold Spring Harb Perspect Med.

[43] Martinho MS, Nancarrow DJ, Lawrence TS, Beer DG, Ray D (2021). Chaperones and ubiquitin ligases balance mutant p53 protein stability in esophageal and other digestive cancers. Cell Mol Gastroenterol Hepatol.

[44] Cunningham JM, Christensen ER, Tester DJ, Kim CY, Roche PC, Burgart LJ (1998). Hypermethylation of the hMLH1 promoter in colon cancer with microsatellite instability. Cancer Res.

[45] Herman JG, Umar A, Polyak K, Graff JR, Ahuja N, Issa JP (1998). Incidence and functional consequences of hMLH1 promoter hypermethylation in colorectal carcinoma. Proc Natl Acad Sci U S A.

[46] Mehrotra J, Varde S, Wang H, Chiu H, Vargo J, Gray K (2008). Quantitative, spatial resolution of the epigenetic field effect in prostate cancer. Prostate.

[47] Shen L, Kondo Y, Rosner GL, Xiao L, Hernandez NS, Vilaythong J (2005). MGMT promoter methylation and field defect in sporadic colorectal cancer. J Natl Cancer Inst.

[48] Sundar R, Ng A, Zouridis H, Padmanabhan N, Sheng T, Zhang S (2019). DNA epigenetic signature predictive of benefit from neoadjuvant chemotherapy in oesophageal adenocarcinoma: results from the MRC OE02 trial. Eur J Cancer.

[49] Weusten B, Bisschops R, Coron E, Dinis-Ribeiro M, Dumonceau JM, Esteban JM (2017). Endoscopic management of Barrett's esophagus: European Society of Gastrointestinal Endoscopy (ESGE) Position Statement. Endoscopy.

[50] Hauge T, Amdal CD, Falk RS, Johannessen HO, Johnson E (2020). Long-term outcome in patients operated with hybrid esophagectomy for esophageal cancer - a cohort study. Acta Oncol.

[51] Holand M, Kolberg M, Danielsen SA, Bjerkehagen B, Eilertsen IA, Hektoen M (2018). Inferior survival for patients with malignant peripheral nerve sheath tumors defined by aberrant TP53. Mod Pathol.

[52] Umar A, Boland CR, Terdiman JP, Syngal S, de la Chapelle A, Ruschoff J (2004). Revised Bethesda guidelines for hereditary nonpolyposis colorectal cancer (Lynch syndrome) and microsatellite instability. J Natl Cancer Inst.

[53] Weisenberger DJ, Campan M, Long TI, Kim M, Woods C, Fiala E (2005). Analysis of repetitive element DNA methylation by MethyLight. Nucleic Acids Res.

[54] Vedeld HM, Skotheim RI, Lothe RA, Lind GE (2014). The recently suggested intestinal cancer stem cell marker DCLK1 is an epigenetic biomarker for colorectal cancer. Epigenetics.

