# Supplementary material for: Targeted genetic and epigenetic profiling of esophageal adenocarcinomas and non-dysplastic Barrett’s esophagus
Source: Clin Epigenetics. 2022 Jun 14;14:77. doi: 10.1186/s13148-022-01287-7 (PMC9195284; doi:10.1186/s13148-022-01287-7)
Supplement: Supplementary file 1 — Additional file 1: Tables S1, S2, S3, and S4. Detailed list of TP53 mutations identified in BE and EAC samples; Summary of the genetic and epigenetic alterations found in neoadjuvant treatment-naïve and treated EAC patients; Promoter methylation frequencies in normal mucosa adjacent to EAC samples; and summary of clinicopathological characteristics of all included EAC patient samples. [file 13148_2022_1287_MOESM1_ESM.docx]

**Table S1.** *TP53* mutations identified in BE and EAC samples.

| **Lesion** | **Patient** | **Exon** | **Codon** | **Mutation** | **Aminoacid** |
| --- | --- | --- | --- | --- | --- |
| **BE** | 13* | 6* | 213* | CGA → CGG* | Arg → Arg* |
| **EAC** | 2 | 4 | 90 | Ins 1 base | Frameshift |
|  | 7 | 4 | – | Del 53 bases | Frameshift |
|  | 14 | 7 | 245 | GGC → AGC | Gly → Ser |
|  | 16 | 7 | – | Ins 11 bases | Frameshift |
|  | 17 | 5 | – | Del 1 base | Frameshift |
|  | 20 | 7 | 245 | GGC → AGC | Gly → Ser |
|  | 21 | 8 | 278 | CCT → CTT | Pro → Leu |
|  | 36 | 7 | 245 | GGC → AGC | Gly → Ser |
|  | 38 | 4 | 36 | CCG → CAG | Pro → Gln |
|  | 39 | 8 | 282 | CGG → TGG | Arg → Trp |
|  | 41 | 4 | – | Del 13 bases | Frameshift |
|  | 43 | 8 | 278 | CCT→ CTT | Pro → Leu |
|  | 51 | 5 | 136 | CAA → TAA | Gln → STOP |
|  | 52 | 8 | 273 | CGT → TGT | Arg → Cys |
|  | 53 | 8 | 273 | CGT → TGT | Arg → Cys |
|  | 55 | 7 | 245 | GGC → AGC | Gly → Ser |
|  | 56 | 6 | 196 | CGA → TGA | Arg → STOP |
|  | 61 | 8 | 282 | CGG → TGG | Arg→ Trp |
|  | 62 | 6 | 220 | TAT → TGT | Tyr→ Cys |
|  | 72 | 7 | 246 | Ins 3 bp | In-frame indel mutation |
|  | 75 | 7 | 256 | ACA → CCA | Thr → Pro |
|  | 78 | 8 | 266 | GGA → AGA | Gly→ Arg |
|  | 81 | 6 | 211 | ACT → ATT | Thr → Ile |
|  | 83 | 5 | 127 | TCC → CCC | Ser → Pro |
|  |  |  | 128 | CCT → CAT | Pro → His |
|  | 87 | 5 | 175 | CGC → CAC | Arg → His |
|  | 90 | 4 | – | Del 71 bases | Frameshift |
|  | 91 | 6 | 213 | CGA → TGA | Arg → STOP |
|  | 94 | 6* | 213* | CGA → CGG* | Arg → Arg* |
|  |  | 8 | 306 | CGA → TGA | Arg → STOP |
|  | 95 | 6 | 213 | CGA → CAA | Arg → Gln |
|  | 104 | 8 | 282 | CGG → TGG | Arg → Trp |

* Classified as *TP53* wild type

**Table S2.** Summary of clinicopathological characteristics and the genetic and epigenetic alterations found in neoadjuvant treatment naïve and treated EAC patients.

|  | **Neoadjuvant treatment-naïve EAC patients**  **(*n* = 27)** | **Neoadjuvant treated EAC patients**  **(*n* = 81)** | ***p*-value** |
| --- | --- | --- | --- |
| Age (years)  Median (mean)  Range  Tumor (T) stage  T0  T1  T2  T3  T4  *TP53* mutations  Yes  No  *APC* promoter hypermethylation  Yes  No  *CDKN2A* promoter hypermethylation  Yes  No  *MGMT* promoter hypermethylation  Yes  No  *TIMP3* promoter hypermethylation  Yes  No  *MLH1* promoter hypermethylation  Yes  No  MSI status  MSI-H  MSI-L/MSS | 76 (75)  65-82  0 (0%)  6 (22%)  6 (22%)  15 (56%)  0 (0%)  12 (44%)  15 (56%)  21 (78%)  6 (22%)  16 (59%)  11 (41%)  5 (19%)  22 (81%)  20 (74%)  7 (26%)  2 (7%)  25 (93%)  1 (4%)  26 (96%) | 64 (62)  34-78  1 (1%)  10 (12%)  14 (17%)  54 (67%)  2 (2%)  18 (22%)  63 (78%)  46 (57%)  35 (43%)  29 (36%)  52 (64%)  18 (22%)  63 (78%)  32 (40%)  49 (60%)  3 (4%)  78 (96%)  2 (2%)  79 (98%) | 3.4 x 10^-10^  NS  0.045  NS  0.043  NS  0.0034  NS  NS |

NS: non-significant association

**Table S3.** Promoter methylation frequencies for the evaluated genes in normal mucosa adjacent to EAC samples.

| **Gene** | **Normal mucosa**  **(*n* = 108)** |
| --- | --- |
| APC | 13 (12%) |
| CDKN2A | 5 (5%) |
| MGMT | 10 (9%) |
| TIMP3 | 4 (4%) |
| MLH1 | 2 (2%) |

**Table S4.** Summary of clinicopathological characteristics of included EAC patient samples.

|  | **EAC**  **(*n* = 145)** |
| --- | --- |
| Age (years)  Median (mean)  Range  Gender  Male  Female  Location  At or above carina  Distal esophagus  Gastroesophageal junction  Tumor (T) stage  T0  T1  T2  T3  T4  Tumor length (cm)  Median (mean)  Range  Lymph node metastases  Yes  No  Neoadjuvant radio(chemo)therapy  Yes  No | 66 (65)  34-82  121 (83%)  24 (17%)  1 (1%)  49 (34%)  95 (65%)  2 (1%)  26 (18%)  30 (20%)  85 (59%)  2 (1%)  3.1 (3.6)  0.4-11  73 (50%)  72 (50%)  117 (81%)  28 (19%) |
