# Supplementary material for: Targeted genetic and epigenetic profiling of esophageal adenocarcinomas and non-dysplastic Barrett’s esophagus
Source: Clin Epigenetics. 2022 Jun 14;14:77. doi: 10.1186/s13148-022-01287-7 (PMC9195284; doi:10.1186/s13148-022-01287-7)

Figure S1. Literature search flow diagram for selection of candidate genes for analysis. In addition, *MLH1* promoter methylation, reported to be infrequent in EAC, was analyzed in order to relate it to MSI status.

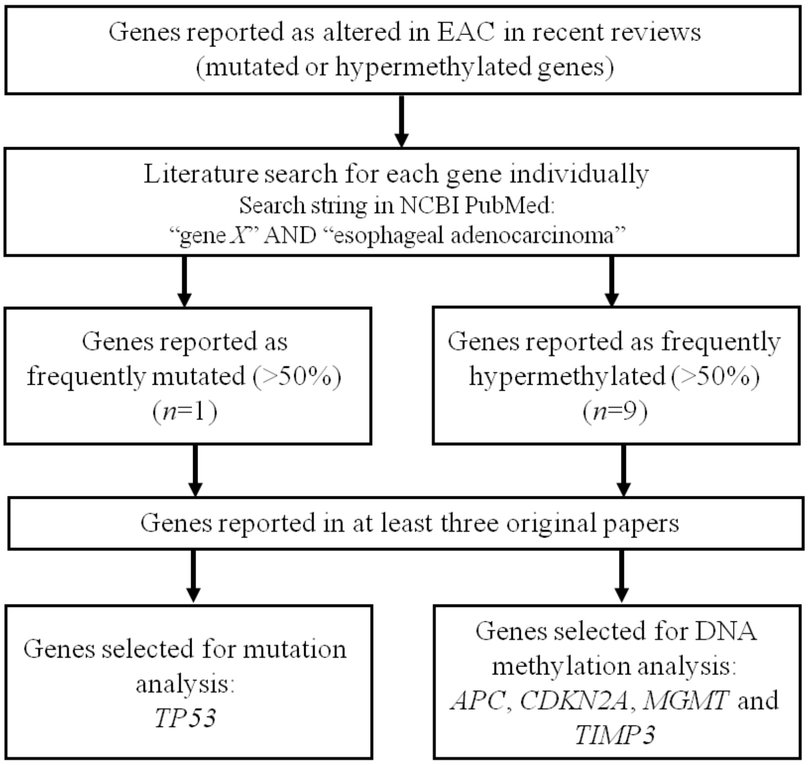

Supplement: Supplementary file 2 — Additional file 2: Figure S1 Literature search flow diagram for selection of candidate genes for analysis. [file 13148_2022_1287_MOESM2_ESM.pdf]
